# Comparative studies for the biotechnological production of l-Lysine by immobilized cells of wild-type *Corynebacterium glutamicum* ATCC 13032 and mutant MH 20-22 B

**DOI:** 10.1007/s13205-015-0275-8

**Published:** 2015-01-24

**Authors:** Meerza Abdul Razak, Buddolla Viswanath

**Affiliations:** 1 Natco Pharma Limited, Natco House, Road No. 2, Banjara Hills, Hyderabad, 500 034 India; 2 Department of Virology, Sri Venkateswara University, Tirupati, 517502 A. P India

**Keywords:** l-Lysine production, *C. glutamicum*, Immobilization, Upstream bioprocess analysis

## Abstract

Establishing a cost and time efficient approach for bioprocess optimization is desired but is challenging. In the present work, we have addressed the effectiveness of using immobilized cells for aerobic processes, behaviour of immobilized cells, optimization and upstream bioprocess analysis for the production of lysine by immobilized cells of *Corynebacterium glutamicum* ATCC 13032 and MH 20-22 B in stirred tank bioreactor. Optimized operational conditions for maximal yield and productivity were determined with six parameters i.e., pH, temperature, fermentation time, airflow rate, glucose concentration and aeration rate. With the obtained results, it was evident that the optimum values for the upstream parameters viz., fermentation time, pH, temperature, glucose concentration, air flow rate and agitation rate are 96 h, 7.5, 30 °C, 90 g/l, 1.0 vvm and 200 rpm for both immobilized cells of *C. glutamicum* ATCC 13032 and MH 20-22 B. Immobilized cells of C*. glutamicum* MH 20-22 B, which is a leucine auxotroph has yielded more l-lysine compare to the immobilized cells of wild type strain *C. glutamicum* ATCC 13032.

## Introduction

Amino acids have been produced with the help of microorganisms for almost 50 years. The economic significance of these cellular building blocks is significant and hence demand is consistently growing and constant efforts to enhance production performance are directed towards the microorganisms, as well as towards technological improvements of the relevant processes. The l-glutamic acid is in first place of highest produced amino acid, followed by l-lysine and dl-methionine whereas the other amino acids follow behind (Anastassiadis 2007). The cause for the increased demand for amino acids stems from their consumption as food preservative, feed supplements, therapeutic agents and precursors for the production of peptides or agrochemicals (Leuchtenberger et al. 2005). l-lysine is essential as a feed additive for poultry and pig breeding.

Gram-positive, rod-shaped bacteria *Corynebacterium glutamicum* has usually occupied a special position within the amino acid producing microorganisms with industrial significance. The essential amino acid l-lysine is one of the most important amino acids applied as supplement in animal feed (Wendisch and Bott 2005; Bercovici and Fuller 2007). The supplementation of such feed materials with a lysine rich source leads to optimized growth of pigs or chicken (Wendisch and Bott 2005). The direct addition of lysine hereby has proven especially valuable. It does not cause an extra uptake and metabolization of other aminoacids beyond their requirement so that superfluous formation of ammonia and environmental burden by increased nitrogen loads in the manure is avoided. The progressing development of an increased utilization of white meat in different countries of the western as well as the eastern world has led to a vast market growth for lysine during the past decades (Tryfona and Bustard 2005). The high importance of lysine in nutrition has inspired intensive research on the lysine, bioprocess optimization, biosynthetic pathways and their regulation and the search for microorganisms capable of over-producing this amino acid. The first steps towards industrial production of lysine were done in Japan in the 1950s when Kyowa Hakko Co., Ltd., Tokyo initiated a research program targeted at finding a microorganim capable to produce glutamate. One of the results from this was the isolation of a microorganism *Micrococcus glutamicus*, later on renamed to *C. glutamicum*, which was able to produce glutamate (Kinoshita et al. 1957; Udaka 1960). During mutagenesis and screening program lysine producing mutants were discovered (Kinoshita et al. 1958) and the basis for lysine production was made. Within a few years the first industrial scale lysine manufacturing facility was developed. Since then lysine fermentation processes have been employed for large scale production. As the research interest for a cost effective production has increased, l-Lysine product prices have decreased. l-Lysine is produced by aerobic fermentation process using the bacterium *C. glutamicum,* it is an aerobic, non-sporulating, gram-positive, bacterium with GRAS (Generally Regarded As Safe) status that has been extensively used for the industrial production of a number of food grade amino acids, feed, and pharmaceutical products for many decades based on classical metabolic engineering. *C. glutamicum* abilities to produce other amino acids like l-threonine (Shiio 1990; Kase and Nakayama 1974; Shiio et al. 1991), l-methionine (Nakayama and Araki 1973; Kalinowski et al. 2003), l-serine (Eggeling 2007), l-histidine (Araki et al. 1974), l-valine (Ruklisha et al. 2007), l-tryptophan (Ikeda 2006), l-phenylalanine and l-tyrosine (Ikeda and Katsumata 1992), l-leucine (Patek 2007) and l-isoleucine (Guillout et al. 2002) has made it major work horse of industrial biotechnology. The biotechnological production of l-lysine by *C. glutamicum* requires a continuous improvement of the lysine production process with a special attention on optimization of the production process and strains engineering (Pfefferle et al. 2003). Therefore, the main objective of this paper is to analyze upstream bioprocess and comparative studies of l-Lysine production by free cells of *Corynebacterium glutamicum* ATCC 13032 and MH 20-22 B in stirred tank bioreactor.

## Materials and methods

The wild-type *C. glutamicum* ATCC 13032 was acquired from American Type and Culture Collection, Manassas, USA and *C. glutamicum* MH 20-22 B was donated by Professor Eggeling, Biotechnology Institute, Julich, Germany. Both these strains were cultured on agar slopes containing peptone (5 g), beef extract (3 g), NaCl (5 g), agar (15 g), distilled water (1,000 ml) was maintained at pH 7.

## Immobilization method

### Growth medium composition

The composition of growth medium is as follows: Glucose (2 g), beef extract (1 g), bacto peptone (1 g), NaCl (0.25 g), agar (2 g), distilled water (100 ml) and the pH 7.

Agar slants of *C. glutamicum* cells which were grown for 24 h were used to inoculate 50 ml of growth media and kept on shaker for 48 h (150 rpm) at 30 °C. Hundred milliliter of 72 h culture was used to prepare immobilized beads of calcium alginate. Fifteen percent volume of beads were employed throughout this study.

Immobilization of *C. glutamicum* cells was done in strict aseptic conditions. Gluteraldehyde entrapment method using cross linked calcium alginate was used to immobilize *C. glutamicum* cells (Jetty et al. 2005; Marek et al. 1985). Hundred microliter gluteraldehyde and 3 % sodium alginate were thoroughly mixed with 0.06 % cells on dry cell weight basis (DCW) (w/v), to get uniform suspension. This uniform suspension was transferred into 0.2 M CaCl_2_ solution using peristaltic pump through a cut micropipette tip (or) orifice. The curing of the formed beads was done by incubating in 0.2 M CaCl_2_ solution for 24 h and washed twice with sterile saline solution [0.9 % NaCl solution (w/v)] and preserved at 4 °C in saline solution for further use. The above immobilization method is followed for both strains of *C. gluatamicum* to get immobilized cells of them.

## Fermentation procedure for lysine production by immobilized cells of *C. glutamicum* ATCC 13032 and MH 20-22 B

Batch or fed-batch processes are employed for the commercial production of amino acids. In batch operations all of the nutrients are added at the beginning. And moreover in batch fermentations microorganisms grows until one or more of essential nutrients get exhausted or until fermentation conditions like oxygen limitation, pH decrease and product inhibition become unfavourable. In the present work fermentation experiments with immobilized cells have been conducted in batch mode using a sterilized stirred tank bioreactor. The immobilized *C. glutamicum* cells were used to inoculate the fermentation medium in the bioreactor and this batch fermentation was carried out for 200 h. The above batch fermentation procedure was carried out at different parameters separately for immobilized cells of both strains, to optimize the upstream parameters. New Brunswick Bioreactor of 5 L was used for the present batch fermentation experiments and was carried out for 120 h. Initially all reactor parts are separated. All parts are washed with distilled water thoroughly and again with acetone before sterilization. After washing with distilled water all the above parts are wrapped with aluminium foil before placing into the autoclave. All these accessories are kept in the autoclave and the autoclave is operated at a temperature of 120 °C and a pressure of 15 psi for a period of 20 min. The aeration rate for the batch fermentation was set to different vvm by the integrated gas flow controller. A pH electrode (Mettler Toledo, Giessen, Germany) and automated addition of 25 % NH_4_OH was used to maintain the pH at 7 in the stirred tank bioreactor. The added volume was determined gravimetrically (Lab Balance Cupis, Sartorius, Gottingen, Germany). Dissolved oxygen was determined using a pO2 (partial pressures of oxygen) electrode (Mettler Toledo, Giessen, Germany) and by variation of the stirrer speed. This was controlled by the process control software Base Lab (BASFSE, Ludwigshafen, Germany).Temperature was maintained at 30 °C using jacket cooling. CO_2_ and O_2_ in the exhaust gas were analyzed by a mass spectrometer. All processes data were monitored online and recorded by BaseLab. The composition of media used for fermentation process in this work is as follow:

CaCl_2_ 2H_2_O (1 g), (NH_4_)2SO_4_ (30 g), MgSO_4_ 7H_2_O (0.4 g), NaCl (0.05 g), MnSO_4_ H_2_O (0.0076 g), FeSO_4_ 7H_2_O (0.001 g), KH_2_PO_4_ (1 g), K_2_HPO_4_ (1 g), Urea (2 g), Yeast extract (1.5 g), Peptone (2 g), d-Glucose (150 g), Thiamine (0.2 mg), d-Biotin (0.5 mg), l-Serine (0.1 mg) and distilled water (1.0 L). And the pH was maintained at 7.0.

## Analytical estimation of l-lysine and substrates

Cell concentration was determined photometrically at 660 nm (UV–Visible spectrophotometer, Thermo Electron Corporation). Supernatant from batch fermentation was used for quantification of substrates and products which were obtained by separation of the biomass by centrifugation at 8,500 g for 5 min at 4 °C. l-lysine was estimated by the method of Chinard (Chinard 1952). Glucose concentration was determined by anthrone method (Morris 1948; Neish 1952). Residual sugar was determined as glucose in the supernatant fluid by the colorimetric DNS Method (Miller 1959). Biomass in the broth was estimated in 1 ml of the sample by centrifugation, and dried in an oven at 105 °C until constant cell weight obtained.

## Results and discussion

Earlier we have carried out many batch fermentations to improve the production rate of l-Lysine by free and immobilized cells of *C. glutamicum* under various process conditions. It was reported from one of our former investigation that l-Lysine can be produced more efficiently by immobilized growing *C. glutamicum* cells compared to free cells in stirred tank bioreactor (Razak and viswanath 2014). In addition, many investigators have stated about the multistage continuous fermentation processes and their difficulties to run continuous l-Lysine production systems (Becker 1982; Michalski et al. 1984). So in the present investigation we have focused on the upstream bioprocess analysis, comparative studies and optimization of l-Lysine production by immobilized cells of wild type *C. glutamicum* ATCC 13032 and *C.glutamicum mutant* MH 20-22 B.

## Effect of fermentation time on l-lysine production by immobilized cells of *C. glutamicum* ATCC 13032 and MH 20-22 B

**Fig. 1:**
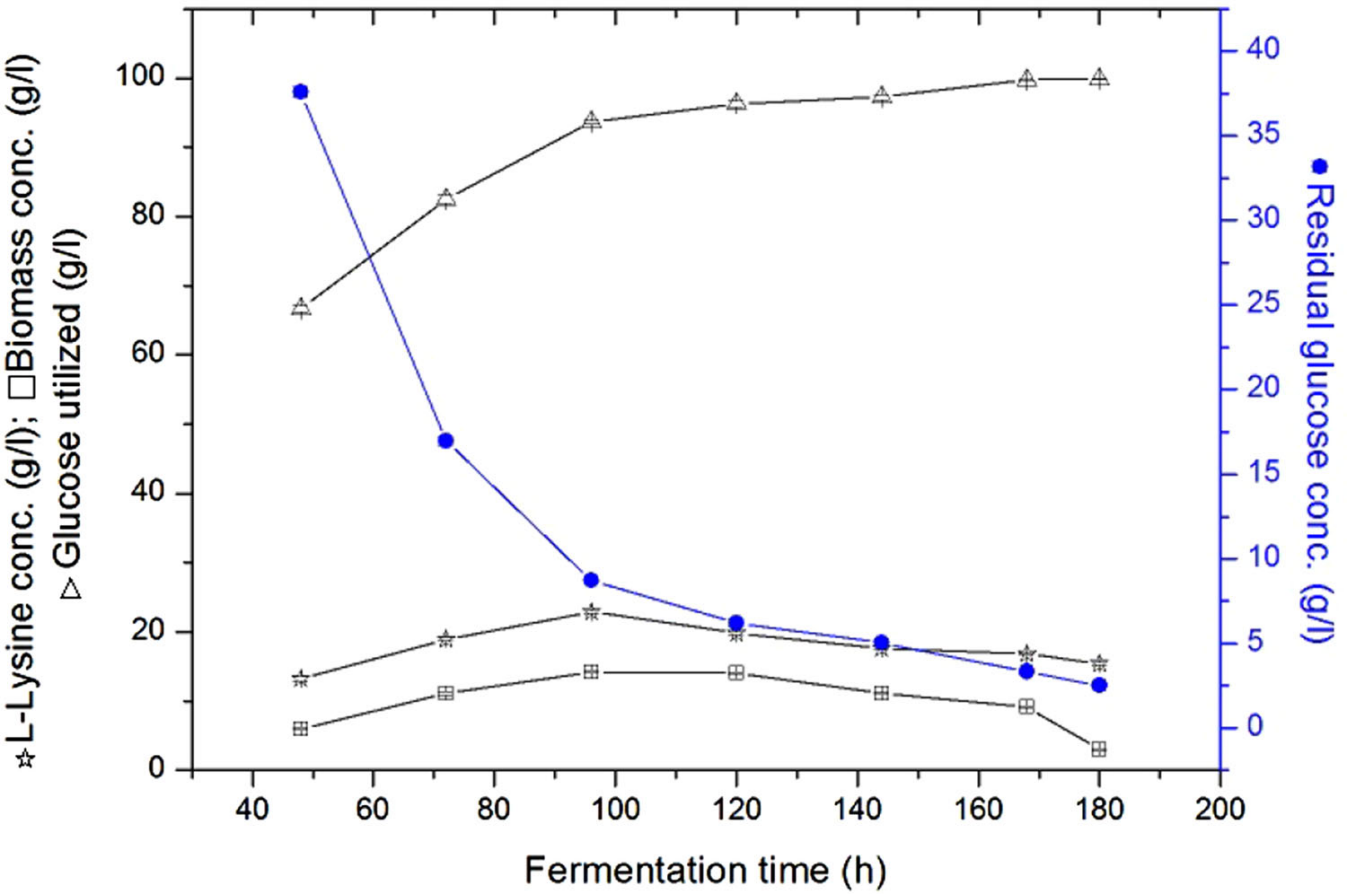
Effect of fermentation time on l-lysine production by immobilized cells of *C. glutamicum* ATCC 13032

The investigation on effect of fermentation time on l-lysine production by immobilized cells of *Corynebacterium glutamicum* ATCC 13032 and MH 20-22 B cells was carried out under operating fermentation conditions like temperature 28 °C, pH 7.0, air flow rate 1.5 vvm, agitation rate of 300 rpm and glucose concentaration of 100 (g/l). It is observed that as a fermentation time increases the concentration of the residual glucose decreases between 40 and 120 h. Similar type of studies was carried out by Amin and Al-Talhi for production of l-Glutamic acid by using immobilized cells of *C. glutamicum* (Amin and Al-Talhi 2007). l-lysine concentration was relatively lower for the first 70 h and the cell were multiplied with rapid growth of biomass. As the fermentation proceeds the glucose consumption and l-lysine production increased at 96 h with high sugar utilization and maximum l-lysine concentration. After 96 h, maximum product achieved a downward trend, l-lysine concentration and yields were found to be decreased; in-spite of an increase in fermentation time due to depletion of nutrients. Figure 1 results describe that the maximum lysine concentration is 22.83 ± 0.21 g/l and biomass concentration is 14.26 ± 0.18 g/l at 96 h for *C. glutamicum* ATCC 13032 immobilized cells.

**Fig. 2:**
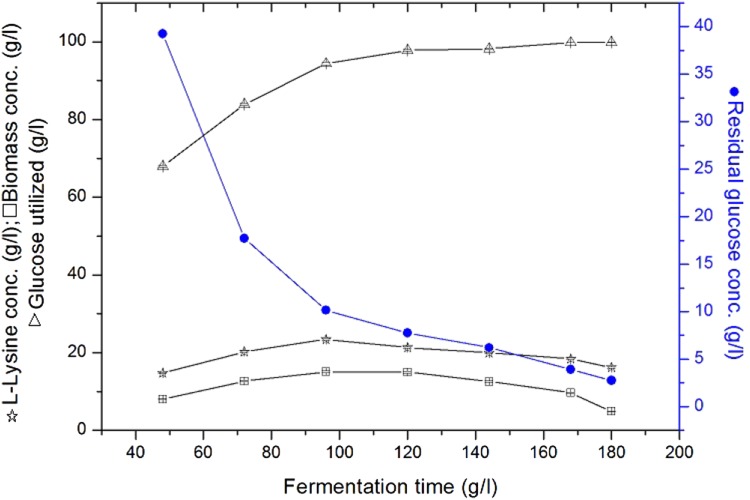
Effect of fermentation time on l-lysine production by immobilized cells of *C. glutamicum* MH 20-22 B

The results presented in the Fig. 2 shows that l-lysine production was more at 96 h, it is the best fermentation time for the production of l-lysine by immobilized cells of *C. glutamicum* MH 20-22 B. The maximal yield of the l-lysine by immobilized cells of *C. glutamicum* MH 20-22 B at 96 h is 23.44 ± 0.27 g/l and biomass concentration is 15.28 ± 0.22 g/l The results presented in the Figs. 1 and 2 shows that l-lysine production was good at 96 h, which is the best fermentation time for the production of l-lysine by immobilized cells of *C. glutamicum* MH 20-22 B.

## Effect of temperature on l-lysine production by immobilized cells of *C. glutamicum* ATCC 13032 and MH 20-22 B

The growth rate of bacterial microorganism is basically dependent on temperature during fermentation which makes changes in whole metabolism. Any change in the temperature can alter the substrate utilization rate of microorganism which leads to unbalanced nutrients in the medium with respect to growth rate of the *C. glutamicum* cells. If any of the crucial nutrients is exhausted soon or unused this can make the culture from balanced to unbalanced growth and results in its performance change. The effect of temperature on l-Lysine production by immobilized cells of *C. glutamicum* ATCC 13032 and MH 20-22 B was studied under different operating conditions like fermentation time of 96 h, pH of 7.0, air flow rate of 1.5 vvm, agitation rate of 300 rpm and glucose concentration of 100 g/l. Glucose utilization was high at 30 °C. The down trend of residual glucose as the temperature increases can be seen after 30 °C the l-lysine biomass concentration was decreased. It was observed that *C. glutamicum* ATCC 13032 immobilized cells had shown maximum l-lysine concentration of 23.03 ± 0.17 (g/l) along with 18.34 ± 0.18 g/l of maximum biomass production at 30 °C (Table 1). Hilliger et al. (1984) observed temperature as one of the key fermentation parameters which has good influence on growth and l-Lysine formation by *C. glutamicum* (Hilliger et al. 1984). An increase in temperature resulted in decreased productivity, which suggested that a little increase in temperature has profound effect on cellular activities, which might be because of repression of metabolic enzymes. *C. glutamicum* MH 20-22 B immobilized cells shows maximum l-lysine production of 24.45 ± 0.19(g/l) and biomass concentration 19.46 ± 0.18 (g/l) at 30 °C (Table 2). The production of l-Lysine by immobilized cells of *C. glutamicum* MH 20-22 B is more when compared with *C. glutamicum* ATCC 13032 immobilized cells.

**Table 1:** Effect of temperature on l-lysine production by immobilized cells of *C. glutamicum* ATCC 13032

Sl. no.	Temperature (°C)	Lysine conc. (p) (g/l)	Biomass (x) (g/l)	Residual glucose conc. (g/l)	Glucose utilized (s) (g/l)
1	27	16.78	13.49	11.12	92.55
2	28	20.22	15.67	7.85	95.88
3	29	21.91	16.00	7.95	96.77
4	30	23.03	18.34	6.17	98.44
5	31	22.56	17.98	3.48	97.65
6	32	19.87	15.90	2.19	95.48

**Table 2:** Effect of temperature on l-lysine production by immobilized cells of *C. glutamicum* MH 20-22 B

Sl. no.	Temperature (°C)	Lysine conc. (p) (g/l)	Biomass (x) (g/l)	Residual glucose conc. (g/l)	Glucose utilized (s) (g/l)
1	27	17.89	15.15	12.33	89.76
2	28	21.74	15.99	9.64	92.45
3	29	22.33	16.79	8.61	93.48
4	30	24.45	19.45	6.42	95.67
5	31	23.05	18.82	4.66	97.43
6	32	21.74	16.44	2.68	99.41

## Effect of pH on l-lysine production by immobilized cells *C. glutamicum* ATCC 13032 and MH 20-22 B

The pH is very important process parameter strongly influences the microbial fermentation. In fermentation process the pH of the broth decreases due to accumulation of other byproducts. As a result the bacterial growth was cease with affiliated decrease in the yield. Basic compounds such as sodium hydroxide, potassium hydroxide, ammonium hydroxide, calcium carbonate, urea, ammonia and inorganic acid compounds such as phosphoric sulphuric acid are used in controlling pH in l-lysine producing cultures ranging from 5.0 to 8.0. The effect of pH on l-lysine yield was found to be very major and crucial parameter. In the present study, effect of pH on l-lysine production was carried out at the fermentation time of 96 h, temperature of 30 °C, air flow rate of 1.5 vvm, agitation rate of 300 rpm and glucose concentration rate of 100 g/l. Figures 1 and 2 shows the effect of pH on l-Lysine production by immobilized cells of *C. glutamicum* ATCC 13032 and MH 20-22 B. pH of the fermentation processes can also be maintained by adding ammonia water along with small amount of CaCO_3_. (Wang et al. 1991). The optimum pH for *C. glutamicum* ATCC 13032 immbolized cells is 7.5 at which the maximum lysine concentration of 23.98 ± 0.15 (g/l) and biomass concentrarion of 19.19 ± 0.15 (g/l) was observed (Table 3). To maintain optimum pH, reagents like calcium carbonate must be added to the culture medium at the beginning of the fermentation, thus calcium carbonate was used as internal neutralizing agent. The maximum l-lysine concentration of *C. glutamicum* MH 20-22 B immobilized cells is 25.63 ± 0.18 (g/l) and biomass concentration is 20.43 ± 0.21 (g/l), it was obtained at 7.5 pH (Table 4). Above results prove that 7.5 pH is the best suited for more lysine production by immobilized cells of *C. glutamicum* ATCC 13032 and MH 20-22 B.

**Table 3:** Effect of pH on l-Lysine production by immobilized cells of *C. glutamicum* ATCC 13032

Sl. no.	pH	Lysine conc. (p) (g/l)	Biomass (x) (g/l)	Residual glucose conc. (g/l)	Glucose utilized (s) (g/l)
1	6	13.16	13.33	11.93	92.02
2	6.5	18.73	17.29	9.32	95.64
3	7	20.91	18.66	6.10	98.65
4	7.5	23.98	19.19	4.79	99.72
5	8	20.19	16.59	3.89	97.08
6	8.5	18.70	14.40	2.59	96.65

**Table 4:** Effect of pH on l-lysine production by immobilized cells of *C. glutamicum* MH 20-22 B

Sl. no.	pH	Lysine conc. (p) (g/l)	Biomass (x) (g/l)	Residual glucose conc. (g/l)	Glucose utilized (s) (g/l)
1	6	14.69	15.47	12.86	89.23
2	6.5	19.9	18.68	10.44	91.65
3	7	21.78	19.33	7.34	94.75
4	7.5	25.62	20.42	5.60	96.49
5	8	21.71	17.53	4.41	97.68
6	8.5	19.7	15.62	2.68	99.41

## Effect of glucose concentration on l-lysine production by immobilized cells of *C. glutamicum* ATCC 13032 and MH 20-22 B

In bioreactor the cell concentration and glucose concentration plays significant task in the performance of a bioprocess for the production of l-lysine. The immobilized cells use available glucose instantly if the process conditions in the reactor are encouraging. The capability and growth of the cells are dependent on the glucose availability in the reactor. *C. glutamicum* can utilize a variety of carbon sources, such as glucose, fructose, sucrose, and maltose for lysine production. The effect of glucose concentration on the l-lysine yield was expressed on the basis of lysine produced per unit substrate utilized. Effect of glucose concentration on l-Lysine production by immobilized cells of *C. glutamicum* ATCC 13032 and MH 20-22 B was studied by carrying fermentation under conditions like pH 7.5, fermentation time of 96 h, temperature 30 °C, air flow rate of 1.5 vvm, agitation rate of 300 rpm and different ranges of glucose concentrations are 70, 80, 90, 100, 110 and 120 (g/l). From running different batches, it was clearly confirmed and concluded that lysine production is cell-growth associated and the growth of the cells is influenced by glucose concentration. The immobilized cells of *C. glutamicum* ATCC 13032 grown at 90 g/l. of glucose concentration have produced maximum lysine with 25.42 ± 0.23 (g/l) and biomass concentration of 18.34 ± 0.14 (g/l). Glucose concentration on l-lysine production was investigated by Hirose and Shibai (1985). and it was described that higher concentration of glucose inhibited bacterial growth along with low yield Hirose and Shibai 1985. Effect of glucose concentration on l-lysine production by immobilized cells of* C. glutamicum *ATCC is summarized in Table 5. Table 6 clearly illustrates that lysine produced by *C. glutamicum* MH 20-22 B immoblized cells is 26.60 ± 0.17 (g/l) and biomass concentration is of 19.68 ± 0.11 at glucose concentration of 90 g/l. As the glucose concentration is increased simultaneously biomass concentration is also increased. It was also seen in the present experiments any excessive substrate concentration present in the fermentation broth leads to decrease in the product concentration. Hadj Sassi et al. (1988) reported that the early concentration of glucose have impact on the production of l-lysine by *Corynebacterium* Sp. in batch culture and found that the specific production rate was obtained at or above 65 g/l of glucose (Hadj Sassi et al. 1988). It was also observed that pH and substrate concentration has significant effect in comparison to temperature.

**Table 5:** Effect of glucose concentration on l-lysine production by immobilized cells of *C. glutamicum* ATCC 13032

Sl. no.	Glucose concentration (g/l)	Lysine conc. (p) (g/l)	Biomass (x) (g/l)	Residual glucose conc. (g/l)	Glucose utilized (s) (g/l)
1	70	16.59	5.37	3.97	67.74
2	80	20.11	10.76	5.93	76.55
3	90	25.42	18.34	6.22	84.92
4	100	22.21	17.91	10.61	90.25
5	110	19.07	16.60	15.49	96.54
6	120	15.99	15.05	18.98	97.48

**Table 6:** Effect of glucose concentration on l-lysine production by immobilized cells of *C. glutamicum* MH 20-22 B

Sl. no.	Substrate conc. (g/l)	Lysine conc. (p) (g/l)	Biomass (x) (g/l)	Residual glucose conc. (g/l)	Glucose utilized (s) (g/l)
1	70	18.5	7.93	4.61	67.48
2	80	21.10	11.38	6.66	75.43
3	90	26.59	19.68	7.47	84.62
4	100	23.7	18.64	12.33	89.76
5	110	20.10	17.46	16.85	97.24
6	120	17.5	16.64	20.66	99.34

## Effect of airflow rate on l-lysine production by immobilized cells of *C. glutamicum* ATCC 13032 and MH 20-22 B

In submerged cultures, the oxygen availability influences microbial production of amino acids. Therefore the oxygen plays crucial task in regulation of both intermediately metabolism and biomass formation coupled with alteration of l-lysine synthesis. If excess of oxygen is supplied, it may lead to formation of other metabolites like succinic acids and lactic acid. While the low availability of oxygen decrease the targeted product production. To investigate the optimum value of air flow rate for immobilized cells of *C. glutamicum* ATCC 13032 and MH 20-22 B experimental studies were conducted in a stirred tank bioreactor under conditions pH 7.5, fermentation time of 96 h, temperature 30 °C, agitation rate of 300 rpm, glucose concentration of 90 g/l and the aeration rate was maintained at different ranges from 0.25 to 1.5. Tables 7 and 8 showed the characteristics batches of l-lysine fermentation at a range of 0.25–1.5 volume air per volume of medium per minute (vvm). The best air flow rate for l-lysine production by *C. glutamicum* ATCC 13032 immobilized cells is 1.0 vvm at which the lysine concentration is 25.21 ± 0.15 g/l and the biomass productivity was is 19.11 ± 0.19 (g/l) (Table 7). Glucose utililized was 85.56 ± 0.27 g/l at 1.0 vvm. So 1.0 vvm was considered as the optimum airflow rate for *C. glutamicum* ATCC 13032. Wang et al. (1991) worked at 200 rpm for fermentation of l-lysine on rotary shaker and suggested optimum air flow rate of l-lysine production is 1.0 vvm or above 1.0 vvm approximately. Table 8 states that maximum lysine production is 26.82 ± 0.17 (g/l), biomass concentration is 20.93 ± 0.20 (g/l) and glucose utililized is 86.48 ± 0.35 (g/l) for *C. glutamicum* MH 20-22 B immobilized cells. Maximum product concentration, biomass and glucose utilized are good at the air flow rate of 1.0 vvm. Therefore optimum air flow rate for l-lysine production by *C. glutamicum* MH 20-22 B immobilized cells is found to be 1.0 vvm.

**Table 7:** Effect of airflow rate on l-lysine production by immobilized cells of *C. glutamicum* ATCC 13032

Sl. no.	Air flow rate (vvm)	Lysine conc. (p) (g/l)	Biomass (x) (g/l)	Residual glucose conc. (g/l)	Glucose utilized (s) (g/l)
1	0.25	14.37	9.94	10.68	76.23
2	0.5	17.97	13.17	9.11	79.34
3	0.75	20.13	18.07	5.69	84.32
4	1	25.21	19.11	4.87	85.56
5	1.25	24.12	16.54	3.04	87.34
6	1.5	23.41	14.76	2.79	87.00

**Table 8:** Effect of airflow rate on l-lysine production by immobilized cells of *C. glutamicum* MH 20-22 B

Sl. no.	Aeration rate (vvm)	Lysine conc. (p) (g/l)	Biomass (x) (g/l)	Residual glucose conc. (g/l)	Glucose utilized (s) (g/l)
1	0.25	16.97	11.39	12.41	78.68
2	0.5	19.68	14.95	10.35	81.56
3	0.75	21.56	19.09	6.96	85.04
4	1	26.85	20.93	5.26	86.47
5	1.25	25.97	18.37	3.53	88.56
6	1.5	24.44	16.78	3.02	88.97

## Effect of agitation rate on l-lysine production by immobilized cells of *C. glutamicum* ATCC 13032 and MH 20-22 B

In bioreactor, fermentation medium was agitated to provide homogeneity across the vessel. Agitation and aeration in a stirred tank bioreactor always causes foaming. Excess foaming drives the broth out of the bioreactor and contaminates the system rapidly. So optimum aeration and agitation must be operated which minimizes foaming and maximizes the lysine production rate. l-lysine production at different agitation rates of 100, 150, 200, 300, 350, 400 rpm under different parameters like pH 7.5, fermentation time of 96 h, temperature of 30 °C, aeration rate of 1.0 vvm and glucose concentration of 90 g/l were investigated to find out the optimum agitation rate by immobilized cells of *C. glutamicum* ATCC and MH 20-22 B. From Table 9 we can observe that maximum l-lysine concentration is 30.17 ± 0.19 g/l and biomass is 16.97 ± 0.19 g/l at 200 rpm. Therefore optimum agitation rate for high l-lysine production is 200 rpm for immobilized cells of *C. glutamicum* ATCC 13032. Shah et al. (2002) investigated about influence of agitation rate and reported that the optimum range is between 50 and 300 rpm (Shah et al. 2002). Similar studies were carried out by Razak and Viswanath for biotechnological production of l-lysine by stirred tank bioreactor and they reported that optimum agitation rate of l-lysine production is 200 rpm for *C. glutamicum* MH 20-22 B (Razak and viswanath 2014). Table 10 shows that the highest lysine yield is 31.58 ± 0.24 (g/l) and maximum biomass concentration is 17.73 ± 0.18 (g/l) for immobilized cells of *C. glutamicum* MH 20-22 B at 200 rpm.

**Table 9:** Effect of agitation rate on l-lysine production by by immobilized cells of *C. glutamicum* ATCC 13032

Sl. no.	Agitation rate (rpm)	Lysine conc. (p) (g/l)	Biomass (x) (g/l)	Residual glucose conc. (g/l)	Glucose utilized (s) (g/l)
1	100	21.45	11.87	9.37	93.55
2	150	25.10	14.54	7.19	94.95
3	200	30.17	16.97	3.99	96.68
4	250	27.32	15.11	6.14	97.35
5	300	25.98	16.40	5.43	98.85
6	350	23.41	15.31	8.97	97.16
7	400	22.72	13.96	10.20	95.33

**Table 10:** Effect of agitation rate on l-lysine production by by immobilized cells of *C. glutamicum* MH 20-22 B

Sl. no.	Agitation rate (rpm)	Lysine conc. (p) (g/l)	Biomass (x) (g/l)	Residual glucose conc. (g/l)	Glucose utilized (s) (g/l)
1	100	23.63	13.45	10.22	80.87
2	150	26.09	15.79	8.41	83.65
3	200	31.58	17.72	4.07	86.03
4	250	28.49	16.77	7.48	84.16
5	300	27.94	17.39	6.82	83.27
6	350	25.78	16.94	10.67	81.42
7	400	23.33	15.09	11.45	80.64

## Conclusions

In order to improve the production rate of l-lysine, immobilized cell of *C. glutamicum* ATCC 13032 and MH 20-22 B were analyzed under various physical and chemical process parameters. Taken together, the results of this study indicated that the optimum values of fermentation time, pH, temperature, glucose concentration, airflow rate and aeration rate were 96 h, 7.5, 30 °C, 90 g/l, 1.0 vvm and 200 rpm respectively for immobilized cells (Table 11). From the results obtained from this investigation we conclude that the immobilized cells of the *C. glutamicum* mutant MH 20-22 B produced more lysine compared to the wild type *C. glutamicum* ATCC 13032. The present study reveals that the bioreactor studies for the optimization of different physical and chemical parameters for the maximum production of l-lysine can also be extended to the fluidized bed bioreactor. The advantage of the bioprocess based on immobilized cells include enhancing microbial cell stability, allowing continuous process operation and avoiding the biomass—liquid separation requirement. Immobilizing *C. glutamicum* cells is one of the bioprocess engineering approaches for improving biotechnological production of l-Lysine. Based on the present studies further studies may be carried out by genetically modified strains to evaluate physical and chemical process parameters for l-lysine production.

**Table 11:** Optimized fermentation parameters for immobilized cells of *C. glutamicum C. glutamicum* ATCC 1303 and MH 20-22 B

Fermentation conditions	Immobilized cells of *C. glutamicum* ATCC 13032	Immobilized cells of *C. glutamicum* MH 20-22 B
Fermentation time (h)	96	96
pH	7.5	7.5
Temperature (°C)	30	30
Glucose concentration (g/l)	90	90
Airflow rate (vvm)	1.0	1.0
Aeration rate (rpm)	200	200
